# Coexisting Hepatic and Renal Dysfunction During Pulmonary Tuberculosis Treatment: A Case Report

**DOI:** 10.1002/ccr3.72661

**Published:** 2026-05-10

**Authors:** Irfan Malik, Nosheen Farooq, Maheen Sheraz, Bilal Aslam, Umama Alam, Umer Sajid, Fatima Sajjad, Kamil Ahmad Kamil

**Affiliations:** ^1^ University of Lahore Lahore Pakistan; ^2^ University of Lahore Teaching Hospital Lahore Pakistan; ^3^ Continental Medical College Lahore Pakistan; ^4^ Khyber Medical College Peshawar Pakistan; ^5^ Rashid Latif Medical College Lahore Pakistan; ^6^ Internal Medicine Department Mirwais Regional Hospital Kandahar Afghanistan

**Keywords:** ANCA‐associated vasculitis, case report, drug‐induced hepatotoxicity, pulmonary tuberculosis, renal dysfunction

## Abstract

Anti‐tuberculosis therapy may rarely cause concurrent liver and kidney injury, with ANCA positivity suggesting immune involvement. This case highlights the importance of early recognition, treatment modification, and multidisciplinary care to prevent complications and ensure effective tuberculosis management.

## Key Clinical Message

Anti‐TB therapy can rarely cause concurrent liver and kidney injury, possibly immune‐mediated. Early detection and timely treatment adjustment are essential to prevent complications and ensure effective TB management.

**Abbreviations**
ADR: adverse drug reactions
ANCA: anti‐neutrophil cytoplasmic antibodies
CRP: C‐reactive protein
CT: computed tomography
LFT: liver function test
RFT: renal function test
TB: tuberculosis
WHO: World Health Organization

## 1 Introduction

Pulmonary tuberculosis (TB) remains a major global health concern, with an estimated 10.6 million new cases and 1.3 million deaths reported worldwide in 2022, according to the World Health Organization (WHO) [1]. Standard first‐line anti‐TB therapy drugs (pyrazinamide, isoniazid, and rifampin) are highly effective; however, their adverse drug reactions (ADRs), especially drug‐induced hepatotoxicity, can become a significant challenge in treatment adherence and success [2]. Co‐occurrence of both hepatic and renal injury during TB treatment is rare and poses significant management challenges, particularly in resource‐limited settings [3]. Additionally, the presence of anti‐neutrophil cytoplasmic antibodies (ANCA) in TB patients is uncommon and may indicate an underlying autoimmune process or vasculitis, further complicating diagnosis and treatment [4]. Here, we present a case of pulmonary tuberculosis complicated by concurrent drug‐induced hepatitis, renal impairment, and ANCA positivity—highlighting diagnostic complexities, therapeutic modifications, and the importance of multidisciplinary management.

### 1.1 Case History

A 46‐year‐old female initially presented with a history of 3 months persistent non‐productive cough. She was treated empirically by a general practitioner with oral antibiotics for 7 days, which failed to produce clinical improvement. Despite the lack of response, no further imaging or laboratory investigations were pursued, and she was advised to continue observation.

Subsequently, a second physician was consulted after 3 weeks due to worsening symptoms, including systemic signs such as elevated C‐reactive protein (CRP). She received a total of 10 intramuscular injections; following the final dose, she developed acute‐onset, symmetrical polyarthralgia, mainly involving the knees, ankles, and wrist which significantly impaired her mobility.

### 1.2 Investigations and Differential Diagnosis

**FIGURE 1:**
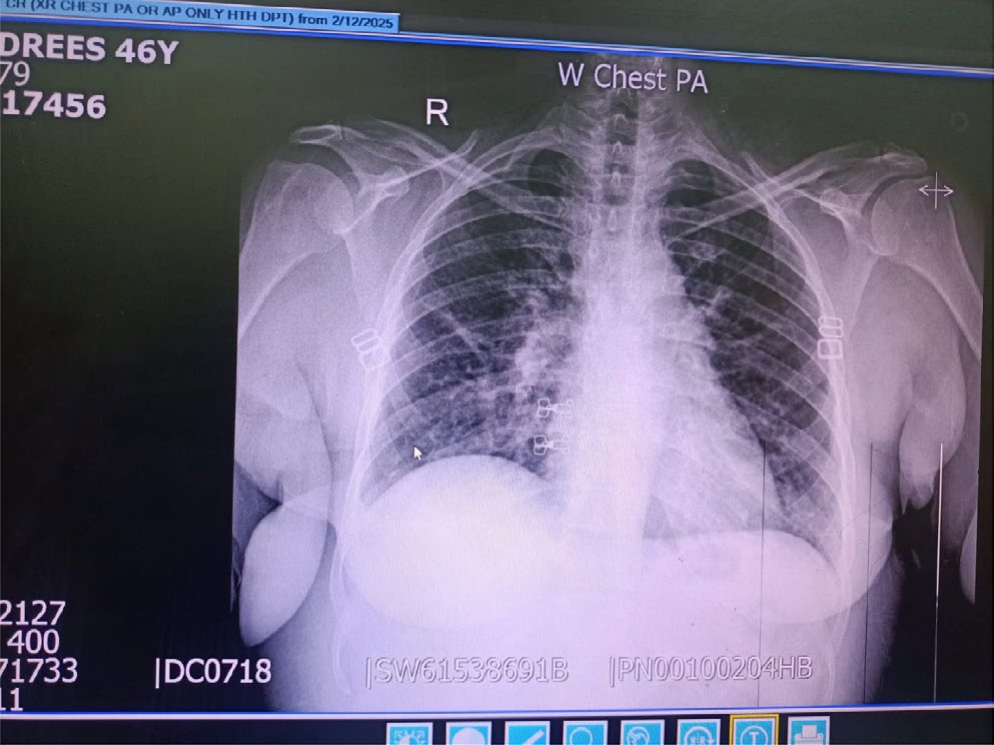
CT scan of the chest.

On December 25, 2024, the patient presented to the University of Lahore (UOL) and was admitted for further evaluation. Vital signs were stable on admission. Investigations included chest radiograph showing right upper lobe consolidation with cavitation, abdominal ultrasound without hepatosplenomegaly, and contrast‐enhanced CT scan of the chest revealing bilateral nodular opacities and mediastinal lymphadenopathy (Figure 1).

Bronchoscopy with bronchoalveolar lavage was performed. Hence, she was diagnosed with pulmonary tuberculosis (TB).

**FIGURE 2:**
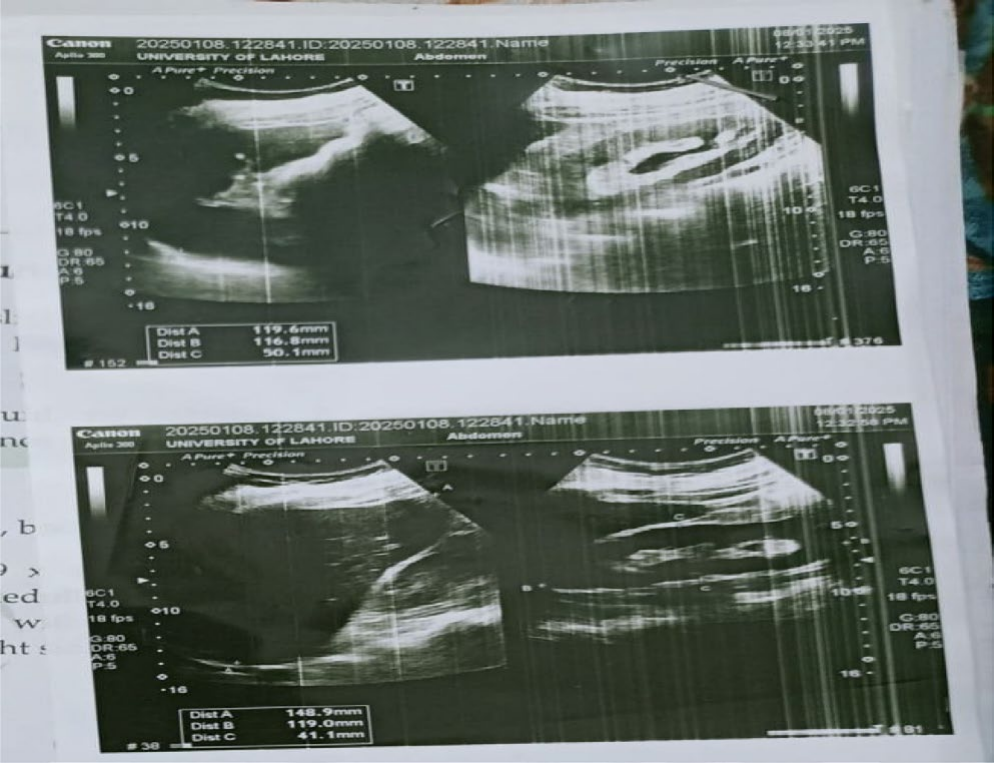
Abdominal ultrasound.

The patient was discharged after 3 days but returned shortly afterward with acute abdominal pain. A repeat abdominal ultrasound was conducted, revealing liver parenchymal changes with slightly increased periportal echogenicity, without focal mass lesion, biliary dilatation, or portal vein abnormality. The gallbladder contained clear fluid with no calculi but showed mild wall thickening (3.8 mm) without pericholecystic fluid. The spleen was normal in size, and the pancreas appeared normal. Both kidneys were of normal size and echotexture, with preserved corticomedullary differentiation; however, the left kidney demonstrated fullness of the pelvicalyceal system despite an empty bladder, and no focal mass lesion, calculus, or hydronephrosis was noted on the right side. No abdominopelvic ascites were detected. Anti‐TB treatment was initiated but poorly tolerated, and within 1 week, she developed vomiting, palpitations, loss of appetite, and poor oral intake. Initial liver function tests (LFTs) and renal function tests (RFTs) were unremarkable, and clinical jaundice was not detected at that time (Figure 2).

In February 2025, she was re‐admitted with new‐onset jaundice and elevated serum creatinine levels (2.4 mg/dL). Anti‐TB medications were withheld, and supportive therapy for hepatic and renal dysfunction was started. Total bilirubin peaked at 5.8 mg/dL and normalized over 16 days. Dialysis was considered but not initiated.

Further investigations revealed that the patient was positive for anti‐neutrophil cytoplasmic antibodies (ANCA). A renal biopsy was advised to investigate possible autoimmune or vasculitic renal involvement; however, the procedure was not performed.

### 1.3 Outcome and Follow‐Up

A modified anti‐TB regimen with reduced hepatotoxicity was initiated, and treatment for both jaundice and kidney dysfunction was continued. At 6‐month follow‐up, the patient exhibited significant improvement in respiratory symptoms—including resolution of cough and dyspnea—indicating good TB control. However, renal impairment persisted, requiring continued management. A renal biopsy remained pending at the time of this report.

## 2 Discussion

This case illustrates the rare coexistence of pulmonary tuberculosis, drug‐induced hepatic injury, renal dysfunction, and ANCA positivity. While hepatotoxicity is a well‐recognized adverse effect of first‐line anti‐TB therapy, renal injury is less common and may arise from diverse mechanisms including drug‐induced interstitial nephritis, glomerulonephritis, or direct infection‐related injury. The concurrent development of hepatic and renal impairment in this patient posed significant diagnostic and therapeutic challenges.

Hepatotoxicity during TB treatment is most frequently associated with isoniazid, rifampicin, and pyrazinamide, with reported incidence rates ranging from 5%–28% depending on geographic region, comorbidities, and genetic predisposition [2, 5]. Risk factors include older age, female sex, malnutrition, viral hepatitis co‐infection, and polypharmacy [6]. In this case, baseline liver function was normal, and hepatotoxicity developed after initiation of standard four‐drug therapy, suggesting a causative role of anti‐TB drugs. Timely recognition and withdrawal of the offending agents, followed by introduction of a modified regimen with reduced hepatotoxic potential, facilitated hepatic recovery [7].

Renal dysfunction during TB therapy is less frequently reported, with etiologies including rifampicin‐induced acute interstitial nephritis, immune‐mediated injury, or, rarely, TB‐associated renal vasculitis [8]. The detection of ANCA in our patient raises the possibility of drug‐induced ANCA‐associated vasculitis (AAV), a rare but documented complication of rifampicin and other antimicrobials [9]. While the absence of renal biopsy precluded histopathological confirmation, the temporal association with drug exposure and persistence of renal impairment despite TB control supports an immune‐mediated mechanism [9].

The presence of ANCA in TB patients has also been described independent of drug exposure, possibly reflecting cross‐reactivity between mycobacterial antigens and host neutrophil proteins [9]. Differentiating between TB‐associated autoantibody production and true drug‐induced vasculitis is clinically important, as the latter may require immunosuppressive therapy [10]. However, in TB patients, immunosuppression must be approached cautiously to avoid disease reactivation.

Although the abdominal ultrasound revealed mild liver parenchymal changes, gallbladder wall thickening, and left pelvicalyceal system fullness, these findings were nonspecific and did not establish a definitive etiology for the patient's subsequent hepatic and renal dysfunction. The absence of pericholecystic fluid, biliary obstruction, or significant renal structural abnormality limited the ability of imaging alone to explain the clinical deterioration. This underscores the need for integrating imaging with laboratory trends and clinical assessment rather than relying solely on sonographic findings.

This case underscores the importance of vigilant monitoring of hepatic and renal function during TB therapy, particularly when symptoms such as jaundice, gastrointestinal intolerance, or reduced urine output emerge. Early multidisciplinary involvement—including infectious disease specialists, nephrologists, and hepatologists—is essential for optimizing outcomes. The case also highlights the need for further research into the immunological interplay between TB infection, anti‐TB drugs, and autoantibody production.

## 3 Conclusion

This case highlights the rare co‐occurrence of drug‐induced hepatotoxicity, renal dysfunction, and ANCA positivity in a patient undergoing treatment for pulmonary tuberculosis. The simultaneous involvement of hepatic and renal systems significantly complicates management, requiring early recognition, prompt drug modification, and close interdisciplinary coordination. Clinicians should maintain a high index of suspicion for ADRs when new systemic symptoms arise during TB therapy and should consider the possibility of autoimmune mechanisms in cases with ANCA positivity. Thorough monitoring and individualized treatment adjustments are essential to ensure both TB control and mitigation of organ damage.

## Author Contributions

**Irfan Malik:** conceptualization, supervision. **Nosheen Farooq:** writing – original draft, conceptualization. **Maheen Sheraz:** conceptualization, writing – review and editing. **Bilal Aslam:** project administration. **Umama Alam:** writing – original draft. **Umer Sajid:** investigation. **Fatima Sajjad:** writing – original draft. **Kamil Ahmad Kamil:** conceptualization, funding acquisition.

## Funding

The authors have nothing to report.

## Ethics Statement

The authors certify that they have obtained all appropriate written patient consent forms. In the form, the patient has given his consent for his images and other clinical information to be reported in the journal. The patient understands that his name and initials will not be published, and due efforts will be made to conceal his identity.

## Consent

Written informed consent was obtained from the patient for publication of this case report and any accompanying images. A copy of the written consent is available for review by the Editor‐in‐chief of this journal.

## Conflicts of Interest

The authors declare no conflicts of interest.

## Data Availability

The data that support the findings of this study are available on request from the corresponding author. The data are not publicly available due to privacy or ethical restrictions.
